# Predicting Concussion Outcome by Integrating Finite Element Modeling and Network Analysis

**DOI:** 10.3389/fbioe.2020.00309

**Published:** 2020-04-15

**Authors:** Erin D. Anderson, J. Sebastian Giudice, Taotao Wu, Matthew B. Panzer, David F. Meaney

**Affiliations:** ^1^Department of Bioengineering, University of Pennsylvania, Philadelphia, PA, United States; ^2^Department of Mechanical and Aerospace Engineering, University of Virginia, Charlottesville, VA, United States; ^3^Department of Biomedical Engineering, University of Virginia, Charlottesville, VA, United States; ^4^Department of Neurosurgery, University of Pennsylvania, Philadelphia, PA, United States

**Keywords:** concussion, biomechanics, networks, structural connectivity, graph theory

## Abstract

Concussion is a significant public health problem affecting 1.6–2.4 million Americans annually. An alternative to reducing the burden of concussion is to reduce its incidence with improved protective equipment and injury mitigation systems. Finite element (FE) models of the brain response to blunt trauma are often used to estimate injury potential and can lead to improved helmet designs. However, these models have yet to incorporate how the patterns of brain connectivity disruption after impact affects the relay of information in the injured brain. Furthermore, FE brain models typically do not consider the differences in individual brain structural connectivities and their purported role in concussion risk. Here, we use graph theory techniques to integrate brain deformations predicted from FE modeling with measurements of network efficiency to identify brain regions whose connectivity characteristics may influence concussion risk. We computed maximum principal strain in 129 brain regions using head kinematics measured from 53 professional football impact reconstructions that included concussive and non-concussive cases. In parallel, using diffusion spectrum imaging data from 30 healthy subjects, we simulated structural lesioning of each of the same 129 brain regions. We simulated lesioning by removing each region one at a time along with all its connections. In turn, we computed the resultant change in global efficiency to identify regions important for network communication. We found that brain regions that deformed the most during an impact did not overlap with regions most important for network communication (Pearson's correlation, ρ = 0.07; *p* = 0.45). Despite this dissimilarity, we found that predicting concussion incidence was equally accurate when considering either areas of high strain or of high importance to global efficiency. Interestingly, accuracy for concussion prediction varied considerably across the 30 healthy connectomes. These results suggest that individual network structure is an important confounding variable in concussion prediction and that further investigation of its role may improve concussion prediction and lead to the development of more effective protective equipment.

## 1. Introduction

Approximately 1.6–2.4 million people are diagnosed with concussion, or mild traumatic brain injury (mTBI), in the United States annually (Corrigan et al., 2010; Taylor et al., 2017). The incidence of mild TBI is rising sharply, with the number of patients diagnosed with concussion increasing by over 60% from 2007 to 2014 (Zhang et al., 2016). Although most patients recover within 3 months (Alexander, 1995; Ponsford et al., 2000; Kashluba et al., 2004), up to an estimated third of patients have post-concussive complaints persisting more than 6 months after injury (Stulemeijer et al., 2008; Norrie et al., 2010; Hou et al., 2012).

Advanced protective headgear remains a key technology for protecting the brain and reducing the incidence and morbidity of concussion. For example, past work shows that using a superior football helmet model can reduce the risk of concussion by 46% (Rowson et al., 2014). Likewise, current bicycle helmets reduce brain injury risk almost 10-fold (Cripton et al., 2014). One key recent advance in helmet design is the number of computational tools available to designers to understand how the direction, magnitude, and timing of an impact lead to damage in different brain regions (Mao, 2018). Most recently, these tools expanded to include a framework to examine impact attenuation properties of different helmets and optimizing these properties prior to fabricating a helmet prototype (Giudice et al., 2019).

Nearly all computational models used to estimate brain injury risk consider the maximum deformation in the brain, regardless of where it occurs, as the primary metric that correlates to injury risk (Takhounts et al., 2013; Gabler et al., 2018, 2019). While these global deformation metrics are suitable for assessing the severity of head impact, they lack the relationships that link local tissue deformation to pathological or functional deficits. In comparison, investigators focused on deformation within specific areas of the brain that are commonly injured in TBI to better predict injury risk (Kleiven, 2007). In the past decade, though, there has been growing interest in considering the brain as a network (Bullmore and Sporns, 2009) and several studies point to specific brain areas and network features that are critically important in intellectual performance (Kim et al., 2016), age-dependent cognitive decline (Shu et al., 2018; Hinault et al., 2019), and working memory (Román et al., 2017). These approaches are equally valuable for understanding changes in the brain following concussion, as several groups have quantified the difference in brain network features (e.g., global efficiency, path length, clustering coefficient, degree) for concussion (Yuan et al., 2015, 2017a; Dall'Acqua et al., 2017; van der Horn et al., 2017) and moderate to severe TBI (Caeyenberghs et al., 2012, 2013, 2014; Kim et al., 2014; Fagerholm et al., 2015; Hellyer et al., 2015; Königs et al., 2017; Solmaz et al., 2017; Yuan et al., 2017b; Verhelst et al., 2018; Watson et al., 2019). Therefore, instead of estimating concussion risk using a measure of the peak mechanical response of the brain during an impact, this recent work allows us to more directly connect the consequences of a mechanical impact with the resulting changes in brain network architecture.

In particular, global efficiency (GE), which represents how efficiently information is exchanged in the network, has been widely reported to decrease following TBI across injury severities, temporal scales post-injury, and subject ages (Caeyenberghs et al., 2014; Yuan et al., 2015, 2017b; Dall'Acqua et al., 2017) and is associated with cognitive deficits following TBI (Caeyenberghs et al., 2014; Kim et al., 2014; Solmaz et al., 2017). For these reasons, linking the strains that occur throughout the brain with the expected changes on the global efficiency of a network could represent a more specific prediction of concussion incidence. Efforts to merge the brain's mechanical response with the resulting alterations in the network architecture do exist in past studies (Kraft et al., 2012), but these studies used idealized sinusoidal impact pulses and did not have reconstructions of real-life impact loading that could be used to estimate concussion risk. As a result, the link between regions affected by mechanical loading and the regions important for network functioning has not been established.

Here, we examine the utility of estimating injury risk after head impact by considering how an impact can affect the structure and, in turn, the global efficiency of a brain network. We hypothesize that relating an impact to the changes in global efficiency of a brain network will improve the accuracy of concussion prediction relative to methods that rely only on the maximum deformation that occurs anywhere in the brain during impact. We use reconstructed kinematic loading conditions from head impacts experienced by professional football players (Sanchez et al., 2019) to determine regions of high strain. We then simulate lesions to the structural connectivity of healthy subjects to evaluate which regions were critical for maintaining global efficiency. We compare the spatial distribution of high strain regions with regions important for maintaining global efficiency. We develop and compare exposure risk curves based on peak deformation of the brain and based on changes to global efficiency. We also examine how intersubject differences in brain architecture and their effect on global efficiency affect the injury risk curve. Together, this work points to the potential for relating an impact more explicitly to the consequences of brain network function, with the possibility of improving concussion prediction and determining architectures that are more vulnerable to impact.

## 2. Materials and Methods

**Figure 1:**
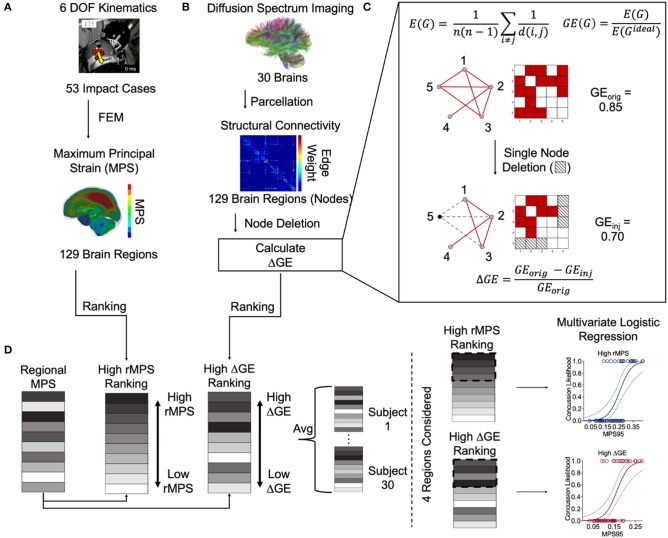
Overview of methods. **(A)** Sanchez et al. estimated the 6 degree-of-freedom kinematics of 53 NFL impact reconstructions and then used them as inputs into a three-dimensional finite element (FE) model developed by Wu et al. (2019b) to estimate the regional maximum principal strain (rMPS) in each of 129 brain regions (Sanchez et al., 2019). Image taken from Sanchez et al. (2019) with permission. **(B)** Betzel et al. parcellated diffusion spectrum imaging from 30 healthy subjects to construct an adjacency matrix representing the structural connectivity between 129 brain regions (Betzel et al., 2016). We performed nodal deletions and then calculated the resultant change in global efficiency (GE). Panel **(C)** is a schematic depicting the equations used for calculating GE itself, the network effects of a simulated single node deletion, and the equation used to calculate the resultant change in GE. **(D)** We ranked the brain regions based on their rMPS and ΔGE. Note that ΔGE is an average of all 30 healthy subject's unique ΔGEs. We reordered the regional rMPSs to match the high rMPS and high ΔGE rankings. Note again that each subject has its own reordered rMPS ranking based on differential ΔGE. We then considered *n* = 1 to 4 regions' rMPS in our multivariate logistic regression, adding rMPSs in order based on the High rMPS Ranking or High ΔGE ranking.

A complete overview of Methods can be found in Figure 1.

### 2.1. Finite Element Modeling

We utilized a three-dimensional finite element (FE) model developed and validated in a previous study (Wu et al., 2019b) to estimate the brain deformation that occurred in response to a defined head impact. Details on the model development and validation can be found in the literature (Wu et al., 2019b). In this study, we do not include axonal fiber strain as has been done by other groups (e.g., Giordano and Kleiven, 2014), and instead focused on the MPS in the gray matter. The prescribed six degree-of-freedom head kinematics were originally reconstructed from helmet-to-helmet impact events occurring in professional football (Newman et al., 1999, 2000, 2005; Pellman et al., 2003), and recently corrected by Sanchez et al. (2019). More information regarding the prescribed head kinematics can be found in Figure S1.

The mesh of the Wu et al. brain model was morphed to the anatomy of the Lausanne brain atlas with 129 regions (Hagmann et al., 2008) using a morphing technique described in Park et al. (2017), in which all elements were morphed using transformations defined by mapping and matching the external geometry of the brains (Wu et al., 2019a,b). For each brain region, the regional maximum principal strain (rMPS) was computed for the elements located within the region. From these, the 95th percentile value was recorded, resulting in a single metric of brain deformation for each of the 129 parcellated brain regions in a given impact case. The global 95th percentile MPS (MPS95) was also calculated, considering all elements in the brain, as is commonly done in FE brain injury analysis to avoid potential numerical instabilities (Panzer et al., 2012; Beckwith et al., 2018; Gabler et al., 2019; Miller et al., 2019; Sanchez et al., 2019; Wu et al., 2019a).

### 2.2. DSI Data Acquisition

Betzel et al. (2016) performed diffusion spectrum imaging (DSI) for a total of 30 subjects along with T1-weighted anatomical scans. Briefly, the DSI data were reconstructed in DSI Studio (http://dsi-studio.labsolver.org) using deterministic fiber tracking until 1,000,000 streamlines had been constructed for each subject. Anatomical scans were segmented using FreeSurfer (Dale et al., 1999) and parcellated using the connectome mapping toolkit (Cammoun et al., 2012). *N*= 129 regions were registered to the B0 volume from each subject's DSI data. From these data, a weighted structural connectivity matrix *C* was constructed wherein the element *C*_*i, j*_ represented the number of streamlines connecting regions *i* and *j*, divided by the sum of the volumes for regions *i* and *j* (Figure S2). The complete list of regions can be found in Table S1.

### 2.3. Network Importance: Lesion Simulation and Global Efficiency Calculation

TBI is frequently characterized as a disconnection syndrome (Guye et al., 2010; Caeyenberghs et al., 2013). However, the widespread damage associated with TBI makes it difficult to pinpoint the specific brain regions most responsible for efficiency of the brain network. As a means to measure the importance of each brain region for network communication more precisely, we systematically lesioned small portions of the network and measured the corresponding effect on global efficiency. The simplest strategy was to delete one node and all its connections, measure the corresponding change in global efficiency, and repeat this single nodal deletion on each of the 129 nodes. Our next, more complex, approach was to randomly delete pairs of nodes and all their connections throughout the brain, again measuring the change in global efficiency for each of these 8256 deletion approaches. Finally, we examined deleting three nodes with their connections simultaneously, and computed global efficiency changes for each of these 349504 deletion strategies. We note that these lesioning approaches were not dependent on strain and were independent from the 53 impact cases. Across our single, pair and triplet node-deletions, the importance of network communication was codified as the normalized change in global efficiency (ΔGE).

Global efficiency is defined as the average inverse shortest path length between two nodes in a network and is calculated as follows using the Brain Connectivity Toolbox (Latora and Marchiori, 2001; Rubinov and Sporns, 2010):

$$\begin{array}{l} E\left(G\right)=\frac{1}{n\left(n-1\right)}\underset{i\neq j\in G}{\sum }\frac{1}{d\left(i,j\right)};\\ GE\left(G\right)=\frac{E\left(G\right)}{E\left(G^{ideal}\right)}, \end{array}$$

where *GE* is the global efficiency, the ratio of the average efficiency (*E*(*G*)) to the global efficiency when all possible edges are present (*E*(*G*^*ideal*^)). The number of nodes in the network is *n* and *d*(*i, j*) represents the length of the shortest path between nodes *i* and *j* in the network *G*.

Global efficiency is a measure of integration, and low global efficiency indicates a greater cost associated with reaching a node from any other node. We selected global efficiency (Bullmore and Sporns, 2009) to evaluate the networks because of its established relevance to clinical TBI data (Caeyenberghs et al., 2014; Yuan et al., 2015, 2017b; Dall'Acqua et al., 2017; Solmaz et al., 2017; van der Horn et al., 2017). To rank the network-efficiency-important regions, we averaged ΔGE across all 30 subjects following each regional deletion. For single-node deletion, we rank-ordered the brain areas based on how much the deletion affected the change in global efficiency. For double and triple node deletions, we first rank-ordered each deletion combination by change in global efficiency, and then weighted by the node's ranking and normalized by the number of times the node appeared in the full list of combinations: 128 for two-node deletions and 8128 for three-node deletions.

### 2.4. Logistic Regression and Cross Validation

Given the binary outcomes of our impacts (concussion or no concussion), we selected logistic regression to evaluate concussion likelihood. To evaluate the efficacy of our method, we used leave-one-out cross-validation (LOOCV) to estimate the accuracy of our prediction of concussion likelihood. LOOCV was selected to maximize our training set for validation given our small dataset size (*n* = 53) and for the low bias that LOOCV exhibits (Beleites et al., 2005). Other studies have employed LOOCV for the reconstructed football impacts and supplemented with out-of-bootstrap cross-validation to address LOOCV's sometimes high variance, but it did not qualitatively alter their findings (Beleites et al., 2005; Cai et al., 2018). We also reported sensitivity and specificity of the cross-validated predictions. Finally, we reported the area under curve (AUC) for the cross-validation testing set using their probability scores and the AUC for the training set. We used no more than 4 predictor variables in our logistic regressions because the 5 events per predictor variable (EPV) rule requires that for the 20 concussion events in our dataset, a maximum of 4 predictors can be used without resulting in biased prediction (Vittinghoff and McCulloch, 2006).

### 2.5. Statistical Analysis

For all comparisons, we used a Wilcoxon Rank-Sum Test at a significance level of 0.05 to compare two conditions, with a Bonferroni correction for *n* = 129 regions where applicable. All plots are shown as mean ± standard deviation.

## 3. Results

**Figure 2:**
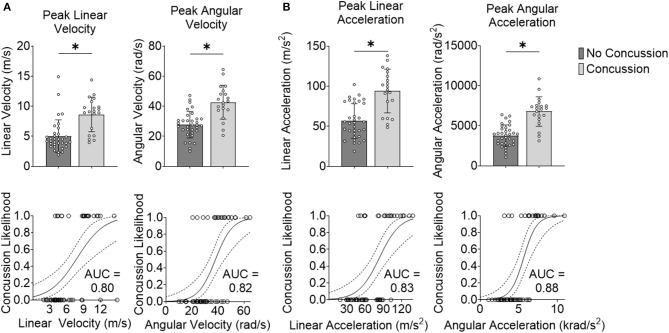
Peak kinematics delineate concussion outcomes. **(A)** Impact velocity is different for concussive vs. non-concussive impacts for peak linear velocity and peak angular velocity. The AUC-testing of the ROC curve for the logistic regression for peak linear velocity was 0.80 and for peak angular velocity was 0.82. **(B)** Impact acceleration is different for concussive vs. non-concussive impacts for peak linear acceleration and peak angular acceleration. The AUC of the ROC curve for the logistic regression for peak linear acceleration was 0.83 and for peak angular acceleration was 0.88. *Indicates significance at the α = 0.05 level; Wilcoxon Rank-Sum Test. Dashed line indicates 95% confidence interval.

Our first step was to test the effectiveness of well-accepted metrics in separating impacts that caused concussion vs. impacts that did not lead to concussion. We observed that the peak resultant kinematics were significantly different between concussion and no concussion cases (*p* = 3.65e-05 for peak linear velocity, *p* = 2.54e-05 for peak angular velocity, *p* = 0.16e-05 for peak linear acceleration, and *p* = 1.01e-06 for peak angular acceleration; Wilcoxon Rank-Sum Test) (Figure 2).

We next investigated the robustness of using these kinematic variables to separate concussion and no concussion cases. For each predictor, we performed logistic regression with leave-one-out cross-validation (LOOCV). We selected LOOCV to validate our model because of its low bias and its suitability for small sample sizes (Beleites et al., 2005). For each predictor, we compared the validation accuracy, sensitivity, specificity, AUC for the testing set, and average AUC for the training sets. For peak resultant velocity and acceleration, univariate logistic regression with LOOCV showed that peak angular acceleration had the highest accuracy (0.85), sensitivity (0.80), specificity (0.88; along with peak linear velocity), testing, and average training AUC (0.88 and 0.90, respectively) (Table 1).

**Table 1:** Peak angular acceleration is the best performing predictor variable.

**Predictor**	**Accuracy**	**Sensitivity**	**Specificity**	**AUC-testing**	**AUC-training average**
Peak linear velocity	0.81	0.70	0.88	0.80	0.84
Peak angular velocity	0.79	0.70	0.85	0.82	0.85
Peak linear acceleration	0.75	0.65	0.82	0.83	0.86
Peak angular acceleration	0.85	0.80	0.88	0.88	0.90
MPS95	0.79	0.70	0.85	0.85	0.87

*Performance of logistic regression classifier using different predictors and leave-one-out cross-validation. Accuracy, sensitivity, specificity and AUC-testing were reported based on the injury prediction of the left out impact case from cross-validation. AUC-training is reported as the average from the training sets*.

**Figure 3:**
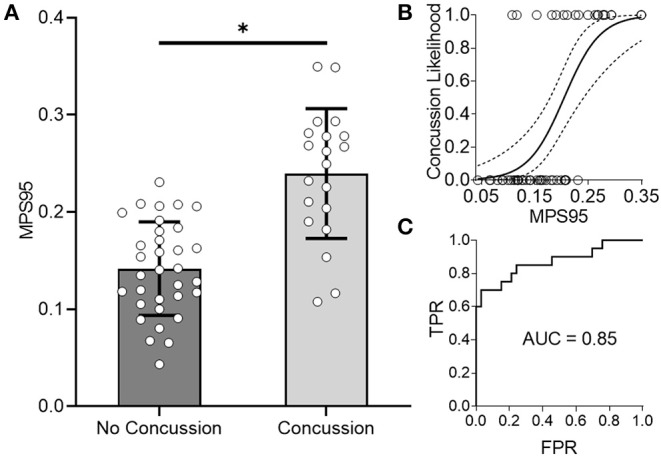
Global 95th Percentile MPS (MPS95) delineates concussion outcomes. **(A)** MPS95 is significantly different for concussion vs. no concussion cases (*p* = 7.24e-06; Wilcoxon Rank-Sum Test). **(B,C)** Univariate logistic regression using MPS95 produces an AUC-testing of 0.85. *Indicates significance at the α = 0.05 level; Wilcoxon Rank-Sum Test.

As an alternative to using loading kinematics for predicting injury risk, we next examined if global 95th percentile maximum principal strain (MPS95) predicted by the FE brain model during impact improved the prediction of concussion likelihood. To evaluate how effectively MPS95 separated concussion and no concussion outcomes, we extracted the peak 95th percentile MPS that occurred at any location in the brain for each of the reconstructed impact loading conditions. As two separate groups, we found a significant difference between concussive and non-concussive impacts' MPS95 (*p* = 7.24e-06; Wilcoxon Rank-Sum Test) (Figure 3A). Univariate logistic regression (Figure 3B) showed that MPS95 had a validation accuracy of 0.79. The logistic regression produced an AUC of 0.87, Figure 3C), sensitivity of 0.70, specificity of 0.85, testing AUC of 0.85, and average training AUC of 0.87, never outperforming peak angular acceleration (Table 1).

**Figure 4:**
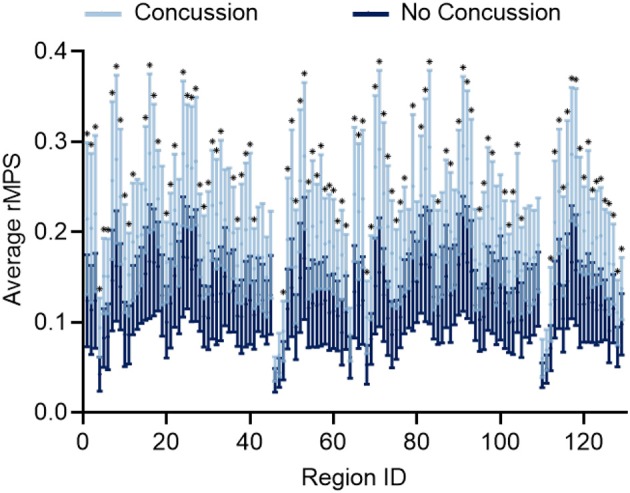
Regional MPS (rMPS) is significantly different for no concussion vs. concussion cases. rMPS is significantly different in concussion vs. no concussion impact cases in 102 regions (*p < 0.05, Wilcoxon Rank-Sum Test with Bonferroni Correction for *n* = 129 comparisons). Error bars represent standard deviation across 20 concussion cases and 33 no concussion cases.

After considering MPS95 as a predictor for concussion, we increased the complexity of our analysis and considered the regional MPS (rMPS) within each of 129 separate brain regions. First, we identified which regions experienced significantly different strain values in concussive vs. non-concussive impacts. For 102 out of 129 regions, there was a significant (α = 0.05, Bonferroni correction for *n* = 129; Wilcoxon Rank-Sum Test) difference in rMPS for concussive vs. non-concussive impacts (Figure 4). Based on historical evidence linking the brain deformation to injury risk (McAllister et al., 2012; Patton et al., 2013), we identified the ten regions that experienced the highest rMPS in impacts causing a concussion (Table 2). These regions were primarily located in the lateral cortex.

**Table 2:** High rMPS regions concentrate in lateral cortex.

**Ranking**	**High rMPS regions**
1	LH postcentral 3
2	LH precentral 4
3	LH pars opercularis 1
4	LH superior temporal 1
5	RH postcentral 1
6	RH precentral 1
7	LH superior temporal 2
8	RH superior temporal 1
9	LH supramarginal 1
10	RH supramarginal 2

*Top 10 regions that experience high strain. RH, right hemisphere; LH, left hemisphere*.

**Figure 5:**
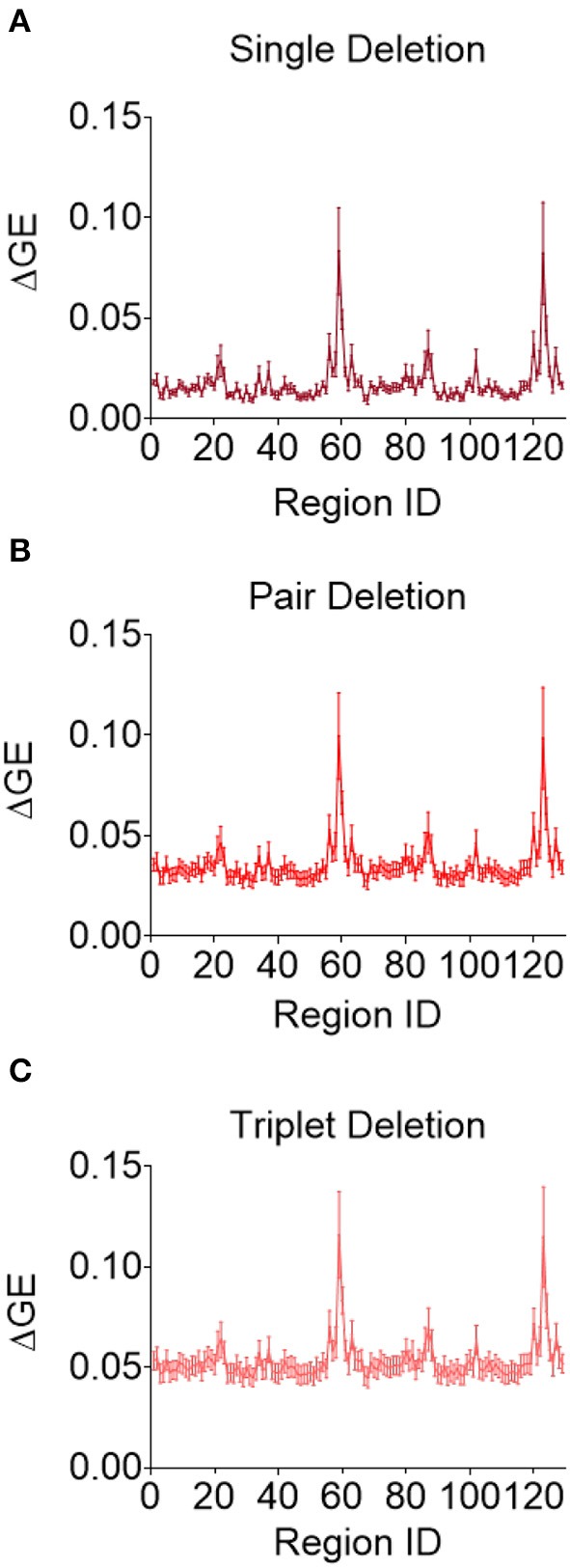
Change in global efficiency after regional deletion is consistent across number of regions deleted. Average change in global efficiency after deletion of one **(A)**, two **(B)**, and three **(C)** brain regions at a time.

Although this analysis tells us which brain regions are more likely to experience damage during injury, it does not provide any inference on how deformation throughout the brain affects network function. To address this shortcoming, we first created synthetic injuries to the network and computed the impact of lesioning an individual region on the overall network performance. As expected, the relative change in global efficiency of the network after deleting each of the 129 nodes (Figure 5A) varied between 0.01 and 0.08. We also considered a more complex injury pattern by deleting pairs and triads of nodes (Figures 5B,C) within the network and found the brain regions that caused the most significant change in global efficiency were nearly identical across these three types of lesion deletion approaches. The top 10 regions for a single node deletion can be found in Table 3. Interestingly, we observed no correlation between the brain regions that showed the largest deformations (Table 2) and the brain areas that were most important for network function (Table 3; *p* = 0.45; ρ = 0.07, Pearson Correlation).

**Table 3:** Regions that produce a high change in global efficiency are concentrated in subcortex.

**Ranking**	**High ΔGE regions**
1	RH caudate
2	LH caudate
3	RH putamen
4	LH putamen
5	LH insula 1
6	RH insula 1
7	LH posterior cingulate 1
8	RH hippocampus
9	LH hippocampus
10	LH pericalcarine 1

*Top 10 regions that experience the greatest change in global efficiency when removed from the network. RH, right hemisphere; LH, left hemisphere*.

**Figure 6:**
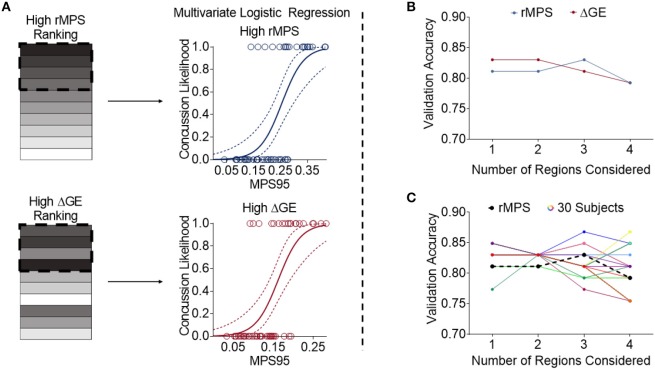
High rMPS and high average ΔGE rMPS are similarly accurate, but there are large differences when considering individual brain architectures. **(A)** The rMPSes corresponding to the High rMPS Regions and the High ΔGE Regions were used for Multivariate Logistic Regression using either 1, 2, 3, or 4 regions at a time. **(B)** When considering the rMPS in multiple regions for logistic regression, adding regions based on ΔGE ranking produced qualitatively similar results compared to adding regions based on High rMPS ranking. **(C)** When considering the individual ΔGE rankings for the 30 healthy subjects, there was considerable variability – up to a 0.12 difference in validation accuracy depending on how many regions' rMPS were considered.

We then took the two different rankings of important brain regions reported in Tables 2, 3 and developed multivariate logistic regressions for predicting concussion outcome. Beginning with the region that showed the largest rMPS in impacts producing concussion, we incrementally added additional areas to the multivariate logistic regression and computed the validation accuracy in each grouping of regions using LOOCV (Figure 6A). We performed the same regression using a progressively larger set of brain regions that were ranked according to their importance in maintaining global efficiency (Figure 6A). In both approaches, we terminated our multivariate regression after 4 regions to avoid overfitting. The regressions produced by adding high ΔGE regions were comparable to those produced by adding high rMPS regions, ranging from 0.75 to 0.83 (Figure 6B, Table 4).

**Table 4:** High rMPS and high average ΔGE rMPS are equivalent predictors.

**Predictor**	**Accuracy**	**Sensitivity**	**Specificity**	**AUC-testing**	**AUC-training average**
High rMPS 1	0.81	0.75	0.85	0.85	0.88
High rMPS 2	0.81	0.70	0.88	0.86	0.89
High rMPS 3	0.83	0.70	0.91	0.84	0.89
High rMPS 4	0.79	0.65	0.88	0.84	0.91
High ΔGE 1	0.83	0.70	0.91	0.85	0.88
High ΔGE 2	0.83	0.75	0.88	0.86	0.90
High ΔGE 3	0.81	0.65	0.91	0.86	0.91
High ΔGE 4	0.75	0.65	0.82	0.83	0.90

*Performance of logistic regression classifier using different predictors and leave-one-out cross-validation. Accuracy, sensitivity, specificity and AUC-testing were reported based on the injury prediction of the left out impact case from cross-validation. AUC-training is reported as the average from the training sets*.

Finally, given that our high ΔGE ranking was based on the average ΔGE across 30 subjects, we wanted to know if the validation accuracy varied across individual subjects' brain architectures. We selected the regions specific to each subject that produced the greatest change in global efficiency. We used the rMPS in those regions to construct a logistic regression to predict concussion incidence across all impacts. We found considerable variability in the validation accuracy across brain architecture, ranging from 0.75 to 0.87 (Figure 6C, Table S2). This variability was also reflected in other measures of predictive power—sensitivity ranged from 0.60 to 0.80, specificity ranged from 0.81 to 0.94, testing AUC ranged from 0.82 to 0.88, and average training AUC ranged from 0.85 to 0.95 (Table S2). The upper bound of this range meets or exceeds that of peak angular acceleration (Table 1), indicating that brain architecture may influence concussion risk.

## 4. Discussion

In this study, we were interested in investigating the role that brain network features play in predicting concussion risk from a head impact. We found that using regional maximum principal strains (rMPSs) in brain regions that are important for network function showed the same prediction accuracy as the approach that used peak strains anywhere throughout the brain (MPS95). Both approaches outperformed peak angular acceleration on the prediction specificity. Finally, our work shows that the accuracy in predicting concussion risk could change as the brain network architecture varies. Although these findings suggest that, on average, the consideration of brain architecture in predicting concussion does not substantially improve the prediction accuracy over many past methods, our results show that individual brain architecture may strongly affect the likelihood of concussion to a given impact.

Our work is one of few to integrate both impact biomechanics and brain network properties to understand how rapid head motions can lead to underlying structural changes to the brain. The first paper, Kraft et al. (2012), simulated a single injury to a representative structural network and examined how global and local efficiency decreased with time after injury. Finding that local efficiency was more affected than global efficiency, this group concluded that the modular nature of the brain helped to prevent loss of efficiency on the global scale from damage at the local scale. In contrast, in our work, we focused on how strain in regions important for global efficiency affected concussion prediction rather than the time course of injury. We found that average local efficiency values significantly correlated with global efficiency, and that 3 out of the top 4 regions for local efficiency matched those for the average change in global efficiency. As a result, we focused our analysis on global efficiency. Furthermore, we expanded on the number of cases, considering the effect of 53 impacts in regions important for 30 subjects rather than one impact and one brain architecture.

In our simulated lesions, we qualitatively reproduced changes observed in concussed structural networks. The regions that produced a large change in global efficiency when removed from the network align well with regions commonly affected in concussion and TBI: hippocampus, posterior cingulate, thalamus, caudate nucleus, insula, temporal cortex (Sharp et al., 2011; Hulkower et al., 2013; Kim et al., 2014; Dall'Acqua et al., 2016). Additionally, regions producing a large change in global efficiency have high betweenness centrality (participate in a large number of shortest paths) and can be considered hub regions, which are commonly implicated in brain disorders (Crossley et al., 2014). These qualitative findings emphasize the importance of networks' (and global efficiency's) role in concussion and TBI.

Furthermore, our findings also reflect what is found in the head impact biomechanics literature. Concussion is widely understood to be an injury caused by rotational motion, with high angular acceleration often cited as the primary biomechanical basis of diffuse brain injury (Ommaya and Gennarelli, 1974; Gennarelli et al., 1982; Meaney et al., 1995; Namjoshi et al., 2014). Considering that MPS95 is highly correlated with angular acceleration in our dataset (ρ = 0.83, Pearson Correlation), it is unsurprising that they had similar prediction accuracy. Kleiven (2007) found using the KTH isotropic finite element (FE) model and the original NFL impact reconstructions that rotational kinematics were the most important factor for intracranial deformations. Furthermore, Beckwith and colleagues found that using MPS across the whole brain, cerebrum, cerebellum, brainstem and corpus callosum were no better than angular acceleration in predicting concussion likelihood for football impacts (Beckwith et al., 2018). In light of their finding and our simple impact events, it is unsurprising that we found that angular acceleration generally outperformed any other predictor, MPS or kinematic parameter.

Our work is not the first to analyze this professional football dataset. King et al. (2003) used the dataset to make the case for using the product of strain and strain rate in the midbrain to predict concussion likelihood. Similarly, Kimpara and Iwamoto (2012) used the dataset to develop criteria for TBI based on angular acceleration, and validated their criteria using the cumulative strain damage measure (CSDM) based on a strain threshold of 15%. Meanwhile, Kleiven (2007) used the KTH isotropic FE model to examine the strain in different brain regions, including the midbrain, brainstem, and thalamus, and the relationships between different injury predictors. Later, Giordano et al. (2017) used the KTH anisotropic FE model to compare the performance of regional maximum axonal strain (rMAS) against rMPS in different brain regions. Zhao et al. (2017) extended Giordano and Kleiven's work using rMAS and sampled across all deep white matter regions of interest and neural tracts to determine regional vulnerabilities. We used an alternative approach that focused on the nodes in the brain, rather than the white matter connectivity (edges) in the network, because this approach would provide a more global measure of the predicted effect on the brain network performance. More recently, Sanchez et al. (2019) updated and corrected the reconstructions of the original NFL dataset (Newman et al., 1999, 2000, 2005; Pellman et al., 2003), which has since been employed in this work and in Wu S. et al. (2019), which utilized yet another approach: developing a neural network to rapidly determine rMPS for injury risk.

However, it has been long known that similar impacts often produce heterogeneous outcomes, and angular acceleration may not be a universal metric for predicting concussion incidence (Bohnen et al., 1992; Beckwith et al., 2013). In more complex, less separable cases than those available to us here, more information about the subject might aid in prediction. Several studies report that neither head kinematics nor impact location were associated with symptom severity and suggest that there could be individualized injury tolerances that govern injury response (Broglio et al., 2011; Rowson et al., 2018). One possible reason for these individual injury tolerances could be due to differences in white matter connectivity and the pattern of injury. There is considerable variability across structural connectivities, with coefficients of variation ranging from 0 to 1.7 for each edge (Cheng et al., 2012). Given our findings suggesting that individual brain architectures will affect the accuracy of predicting concussion, this well-characterized variability could play a key role in individual concussion risk. It would not be the first neurological disorder to tie risk with brain architecture—e.g., in schizophrenia, a 12.7% reduction in rich club connectivity distinguishes unaffected siblings from schizophrenic siblings (Collin et al., 2013). Given this precedent, we expect subject-specific changes to network connectivity may explain at least a portion of heterogeneous concussion outcomes.

Our work also complements clinical studies on changes in the brain networks of concussed patients. On average, a reduction in global efficiency in TBI patients is associated with worse performance in executive functioning, verbal learning, and processing speed (Kim et al., 2014; Solmaz et al., 2017) and worse switching task performance (Caeyenberghs et al., 2014). An increase in global efficiency following aerobic training was associated with an improvement in post-concussion symptom inventory scores (van der Horn et al., 2017; Yuan et al., 2017b) found that global efficiency was significantly different for concussion patients with post-traumatic complaints relative to concussion patients without complaints. Interestingly, van der Horn et al. found that neither subgroup was significantly different from uninjured controls, but that finding could be due to the small sample size or use of binarized connectivity rather than weighted connectivity. In other neurologic diseases, it has already been shown that network features are predictive of outcomes. For subjects with mild cognitive impairment, network features were predictive of volumetric atrophy in 6 months and conversion to Alzheimer's Disease (Nir et al., 2015; Sun et al., 2019). Furthermore, progressive deterioration of the rich club organization dynamically reflects the progression of Alzheimer's Disease (Yan et al., 2018). Because of the association between networks and outcomes, even if network-informed strain predictors are no better than peak angular acceleration for concussion prediction, they could prove useful for predicting 3- or 6-month outcomes.

How changes in structural connectivity give rise to functional deficits is a topic that is still under active investigation. Although after TBI there is a decrease in structural connectivity owing to the physical disconnection of white matter tracts, there are heterogeneous changes to regional brain activation in functional connectivity (Sharp et al., 2014), and it is unclear how structural deficits produce those functional changes. Hellyer and colleagues have attempted to bridge this gap by using phase-coupled oscillators to estimate functional connectivity from the structural connectivity of TBI patients (Hellyer et al., 2015). They found that the altered structural networks produced functional networks that had reduced metastability, a measure of cognitive flexibility (Hellyer et al., 2015). Their work builds on that of other groups employing Kuramoto oscillator models to study how targeted lesion to structural networks affect functional connectivity (Honey and Sporns, 2008; Alstott et al., 2009; Váša et al., 2015). They find that hub nodes, much like the regions important for network communication identified in this work, have a greater impact on network dynamics when removed from the network (Honey and Sporns, 2008; Alstott et al., 2009; Váša et al., 2015). Understanding how biomechanical trauma affects the structural network could provide valuable insights into functional deficits after TBI.

A second area receiving increasing attention in the literature is the rebuilding of physical connections in the brain over time after TBI. For example, Zhu et al. (2015) found that the structural connectivity of the network was unchanged between 24 h and 30 days after concussion. However, on a longer timescale, Wang et al. (2019) found a deterioration in interhemispheric connectivity between 14 days and 1 year after concussion. Our approach used a simple binary deletion scheme – the node and its connections were completely deleted. A more gradual disconnection scheme to mimic how strain might partially break white matter connections would lead to a more gradual change in global efficiency across impact severity and could be combined with systems level models of brain plasticity to estimate the recovery of connectivity following a given impact. Working within this framework of using strain to inform white matter degradation, one could also use an approach developed by Kraft et al. (2012) to incorporate measured changes in cell death within an organotypic slice culture as a model for connectivity over 72 h post impact. Interestingly, this approach showed the same network effects as applying a simple strain-based threshold to decide whether to delete an edge, suggesting we would conclude the same regions as being important for network communication if we considered the effect of time.

A challenge faced by this study is that we lack subject-specific features other than head kinematics. First, the physical properties of the players are not explicitly accounted for, and these properties among players may affect concussion risk. Head and brain morphology, neck strength, and concussion history play a known role in concussion likelihood, influencing how strains develop in the brain during impact (Guskiewicz et al., 2003; Danelson et al., 2008; Collins et al., 2014). It was not possible to acquire such information based on our historical dataset. However, others have found that when examining how different brains respond to the same loading conditions, the MPS experienced by the brain had a coefficient of variation of 2.33% (Giordano et al., 2017). In our model, we minimized the error introduced by uncertainty in head kinematics by using the most current kinematic loading conditions (Sanchez et al., 2019). Finally, and most importantly, we do not have brain imaging data for the subjects who suffered the impacts. As a result of this limitation, we cannot directly link subject-specific regions important for network communication with their head kinematics. Rather, we can only infer how connectivity could play a role in concussion risk.

Regarding the validity of finite element models, this work relies on the biofidelity of computational models to predict region-specific brain deformation. Unfortunately, experimental data for validating regional brain deformation in the FE models are not sufficient. The brain FE model used in this study was evaluated with experimental brain deformation data, including a subset of the data from recent *in situ* studies (Alshareef et al., 2018). The fidelity of the FE models will be improved with the recent advances in *in situ* experimental studies (Alshareef et al., 2020; Zhou et al., 2020), but the markers used to measure brain deformation in those experiments are still sparse and rarely located in the cortical and subcortical gray matter regions, which are the regions of interest in this study.

Furthermore, an important challenge for concussion research in general is recruiting sufficient subjects to collect data on concussion events. In this particular dataset, the cases have been selected such that the concussion and no concussion cases have little overlap in impact kinematics (namely peak angular acceleration), which means that prediction concussion outcome is uncomplicated. It is possible that for more complicated cases, a more nuanced predictor may outperform angular acceleration. It has been shown that for impacts that have a high level of compliance, which results in long duration impacts, MPS may be a more relevant predictor of concussion than kinematics (Rousseau, 2014). Regardless, because concussion is widely believed to have a predominantly mechanical etiology, kinematics will always play a role as a potential grouping variable. Additionally, because we had a limited number of cases, we were not able to leverage a large number of potential concussion predictors at once and were limited to up to four at a time (Vittinghoff and McCulloch, 2006). It is likely that with more predictors in the model and proper sample size for validation, we could generate an even better concussion predictor and home in on what characteristics are most associated with concussion.

From a larger perspective, our work showing that individual brain architecture influences concussion prediction accuracy implies that each person's connectome affects individual concussion risk after head impact. Equipped with information on each person's unique brain architecture, one could simulate a variety of head impact scenarios and assign an overall risk for each person. For athletes in contact sports, this process would provide a useful screening tool for risk assessment prior to play. Moreover, this analysis may even inform protective equipment designed for individuals, rather than the current universal design approach.

This work constitutes one of few studies to considering the interplay between the network properties of structural connectomes and injury biomechanics. In our work, we found a potential role that individual connectomes might play in governing concussion risk. In the long term, this work highlights the potential importance of investigating the structural connectivities of athletes before and after concussion, in conjunction with recording of head impact exposure, as part of a larger research agenda for mitigating concussions in contact sports.

## Data Availability Statement

The datasets generated for this study are available on request to the corresponding author.

## Author Contributions

EA and DM designed the study. EA analyzed network data and trained and validated the prediction model. JG and TW analyzed strain data. DM and MP supervised this work. EA and DM wrote the manuscript with feedback from JG, TW, and MP.

### Conflict of Interest

The authors declare that the research was conducted in the absence of any commercial or financial relationships that could be construed as a potential conflict of interest.
