# Supplementary material for: Predicting Concussion Outcome by Integrating Finite Element Modeling and Network Analysis
Source: Front Bioeng Biotechnol. 2020 Apr 15;8:309. doi: 10.3389/fbioe.2020.00309 (PMC7174699; doi:10.3389/fbioe.2020.00309)
Supplement: Supplementary file 1 [file Data_Sheet_1.PDF]

frontiers

Supplementary Material

1 SUPPLEMENTARY TABLES AND FIGURES

1.1 Figures

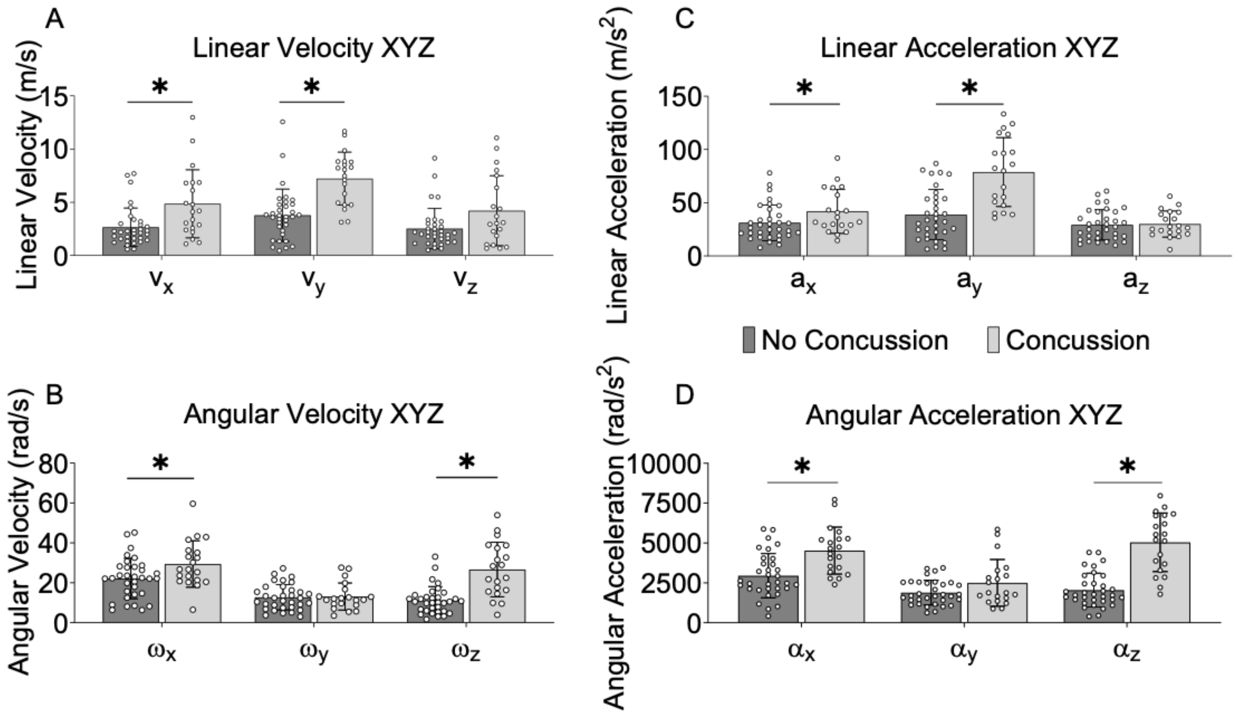

Figure S1. Linear kinematics in x- and y-directions and angular kinematics in x- and z-directions are significantly different for no concussion versus concussion impacts. **(A)** There was a significant difference for linear velocity in the x-direction and y-direction, and **(B)** angular velocity in the x-direction and z-direction. **(C)** There was a significant difference for linear acceleration in the x-direction and y-direction, and **(D)** angular acceleration in the x-direction and z-direction. * indicates significance at the α = 0.05 level; Wilcoxon Rank-Sum Test.

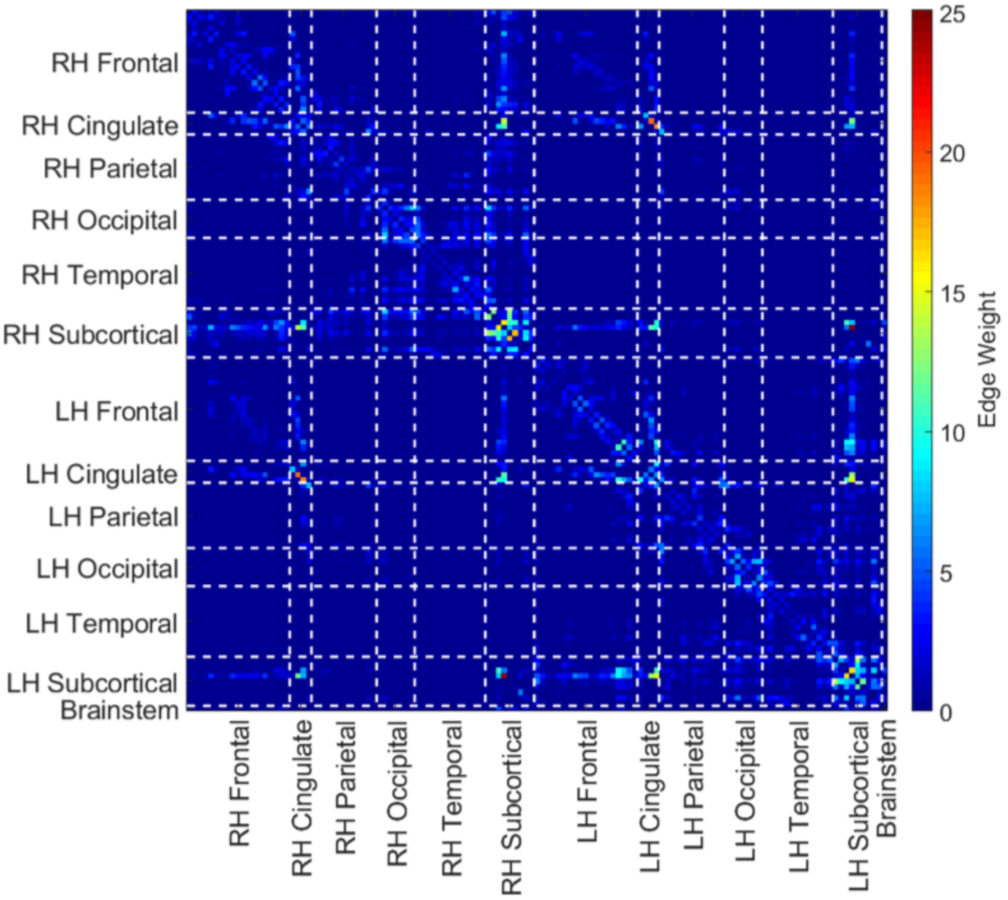

Figure S2. Structural Connectivity. The structural connectivity matrix represents the strength of connection (edge weight) between two brain regions. Regions 1-64 represent the right hemisphere (RH) and regions 65-128 represent the left hemisphere (LH). Region 129 is the brainstem. This connectivity matrix represents one healthy subject.

1.2 Tables

Table S1: **List of brain regions.** Brain regions used for analysis. RH denotes right hemisphere; LH denotes left hemisphere.

Region Number	Region Name	Region Number	Region Name
1	RH Lateral Orbitofrontal 1	65	LH Lateral Orbitofrontal 1
2	RH Lateral Orbitofrontal 2	66	LH Lateral Orbitofrontal 2
3	RH Pars Orbitalis 1	67	LH Pars Orbitalis 1
4	RH Frontal Pole 1	68	LH Frontal Pole 1
5	RH Medial Orbitofrontal 1	69	LH Medial Orbitofrontal 1
6	RH Medial Orbitofrontal 2	70	LH Pars Triangularis 1
7	RH Pars Triangularis 1	71	LH Pars Opercularis 1

Table S1: **List of brain regions.** Brain regions used for analysis. RH denotes right hemisphere; LH denotes left hemisphere.

Region Number	Region Name	Region Number	Region Name
8	RH Pars Opercularis 1	72	LH Rostral Middle Frontal 1
9	RH Rostral Middle Frontal 1	73	LH Rostral Middle Frontal 2
10	RH Rostral Middle Frontal 2	74	LH Rostral Middle Frontal 3
11	RH Superior Frontal 1	75	LH Superior Frontal 1
12	RH Superior Frontal 2	76	LH Superior Frontal 2
13	RH Superior Frontal 3	77	LH Superior Frontal 3
14	RH Superior Frontal 4	78	LH Superior Frontal 4
15	RH Caudal Middle Frontal 1	79	LH Caudal Middle Frontal 1
16	RH Precentral 1	80	LH Precentral 1
17	RH Precentral 2	81	LH Precentral 2
18	RH Precentral 3	82	LH Precentral 3
19	RH Paracentral 1	83	LH Precentral 4
20	RH Rostral Anterior Cingulate 1	84	LH Paracentral 1
21	RH Caudal Anterior Cingulate 1	85	LH Rostral Anterior Cingulate 1
22	RH Posterior Cingulate 1	86	LH Caudal Anterior Cingulate 1
23	RH Isthmus Cingulate 1	87	LH Posterior Cingulate 1
24	RH Postcentral 1	88	LH Isthmus Cingulate 1
25	RH Postcentral 2	89	LH Postcentral 1
26	RH Supramarginal 1	90	LH Postcentral 2
27	RH Supramarginal 2	91	LH Postcentral 3
28	RH Superior Parietal 1	92	LH Supramarginal 1
29	RH Superior Parietal 2	93	LH Supramarginal 2
30	RH Superior Parietal 3	94	LH Superior Parietal 1
31	RH Inferior Parietal 1	95	LH Superior Parietal 2
32	RH Inferior Parietal 2	96	LH Superior Parietal 3
33	RH Inferior Parietal 3	97	LH Inferior Parietal 1
34	RH Precuneus 1	98	LH Inferior Parietal 2
35	RH Precuneus 2	99	LH Precuneus 1
36	RH Cuneus 1	100	LH Precuneus 2
37	RH Pericalcarine 1	101	LH Cuneus 1
38	RH Lateral Occipital 1	102	LH Pericalcarine 1
39	RH Lateral Occipital 2	103	LH Lateral Occipital 1
40	RH Lateral Occipital 3	104	LH Lateral Occipital 2
41	RH Lingual 1	105	LH Lingual 1
42	RH Lingual 2	106	LH Lingual 2
43	RH Fusiform 1	107	LH Fusiform 1
44	RH Fusiform 2	108	LH Fusiform 2
45	RH Parahippocampal 1	109	LH Parahippocampal 1
46	RH Entorhinal 1	110	LH Entorhinal 1

Table S1: **List of brain regions.** Brain regions used for analysis. RH denotes right hemisphere; LH denotes left hemisphere.

Region Number	Region Name	Region Number	Region Name
47	RH Temporal Pole 1	111	LH Temporal Pole 1
48	RH Inferior Temporal 1	112	LH Inferior Temporal 1
49	RH Inferior Temporal 2	113	LH Inferior Temporal 2
50	RH Middle Temporal 1	114	LH Middle Temporal 1
51	RH Middle Temporal 2	115	LH Middle Temporal 2
52	RH Banks of STS 1	116	LH Banks of STS 1
53	RH Superior Temporal 1	117	LH Superior Temporal 1
54	RH Superior Temporal 2	118	LH Superior Temporal 2
55	RH Transverse Temporal 1	119	LH Transverse Temporal 1
56	RH Insula 1	120	LH Insula 1
57	RH Insula 2	121	LH Insula 2
58	RH Thalamus Proper	122	LH Thalamus Proper
59	RH Caudate	123	LH Caudate
60	RH Putamen	124	LH Putamen
61	RH Pallidum	125	LH Pallidum
62	RH Accumbens Area	126	LH Accumbens Area
63	RH Hippocampus	127	LH Hippocampus
64	RH Amygdala	128	LH Amygdala
		129	Brainstem

Table S2: **Intersubject prediction analysis.** Performance using the rMPS in each individual subject's High ΔGE Regions as predictors.

Subject Number & Number of Regions	Accuracy	Sensitivity	Specificity	AUC-Testing	AUC-Training Average
Subject 1 - 1	0.83	0.70	0.91	0.85	0.88
Subject 2 - 1	0.85	0.75	0.91	0.87	0.90
Subject 3 - 1	0.83	0.70	0.91	0.85	0.88
Subject 4 - 1	0.85	0.75	0.91	0.87	0.90
Subject 5 - 1	0.83	0.70	0.91	0.85	0.88
Subject 6 - 1	0.85	0.75	0.91	0.87	0.90
Subject 7 - 1	0.83	0.70	0.91	0.85	0.88
Subject 8 - 1	0.83	0.70	0.91	0.85	0.88
Subject 9 - 1	0.85	0.75	0.91	0.87	0.90
Subject 10 - 1	0.83	0.70	0.91	0.85	0.88
Subject 11 - 1	0.85	0.75	0.91	0.87	0.90

Table S2: **Intersubject prediction analysis.** Performance using the rMPS in each individual subject's High ΔGE Regions as predictors.

Subject Number & Number of Regions	Accuracy	Sensitivity	Specificity	AUC-Testing	AUC-Training Average
Subject 12 - 1	0.77	0.65	0.85	0.82	0.85
Subject 13 - 1	0.81	0.70	0.88	0.86	0.89
Subject 14 - 1	0.83	0.70	0.91	0.85	0.88
Subject 15 - 1	0.83	0.70	0.91	0.85	0.88
Subject 16 - 1	0.85	0.75	0.91	0.87	0.90
Subject 17 - 1	0.85	0.75	0.91	0.87	0.90
Subject 18 - 1	0.83	0.70	0.91	0.85	0.88
Subject 19 - 1	0.85	0.75	0.91	0.87	0.90
Subject 20 - 1	0.83	0.70	0.91	0.85	0.88
Subject 21 - 1	0.85	0.75	0.91	0.87	0.90
Subject 22 - 1	0.83	0.70	0.91	0.85	0.88
Subject 23 - 1	0.83	0.70	0.91	0.85	0.88
Subject 24 - 1	0.85	0.75	0.91	0.87	0.90
Subject 25 - 1	0.83	0.70	0.91	0.85	0.88
Subject 26 - 1	0.83	0.70	0.91	0.85	0.88
Subject 27 - 1	0.85	0.75	0.91	0.87	0.90
Subject 28 - 1	0.85	0.75	0.91	0.87	0.90
Subject 29 - 1	0.83	0.70	0.91	0.85	0.88
Subject 30 - 1	0.85	0.75	0.91	0.87	0.90
Subject 1 - 2	0.83	0.75	0.88	0.86	0.90
Subject 2 - 2	0.83	0.70	0.91	0.87	0.90
Subject 3 - 2	0.83	0.75	0.88	0.86	0.90
Subject 4 - 2	0.83	0.75	0.88	0.86	0.90
Subject 5 - 2	0.83	0.75	0.88	0.86	0.90
Subject 6 - 2	0.83	0.75	0.88	0.86	0.90
Subject 7 - 2	0.83	0.75	0.88	0.86	0.90
Subject 8 - 2	0.83	0.75	0.88	0.86	0.90
Subject 9 - 2	0.83	0.75	0.88	0.86	0.90
Subject 10 - 2	0.83	0.75	0.88	0.86	0.90
Subject 11 - 2	0.83	0.75	0.88	0.86	0.90
Subject 12 - 2	0.83	0.70	0.91	0.83	0.87
Subject 13 - 2	0.81	0.70	0.88	0.85	0.89
Subject 14 - 2	0.83	0.75	0.88	0.86	0.90
Subject 15 - 2	0.83	0.75	0.88	0.86	0.90
Subject 16 - 2	0.83	0.75	0.88	0.86	0.90
Subject 17 - 2	0.83	0.75	0.88	0.86	0.90
Subject 18 - 2	0.83	0.70	0.91	0.84	0.88

Table S2: **Intersubject prediction analysis.** Performance using the rMPS in each individual subject's High ΔGE Regions as predictors.

Subject Number & Number of Regions	Accuracy	Sensitivity	Specificity	AUC-Testing	AUC-Training Average
Subject 19 - 2	0.83	0.75	0.88	0.86	0.90
Subject 20 - 2	0.83	0.75	0.88	0.86	0.90
Subject 21 - 2	0.83	0.75	0.88	0.86	0.90
Subject 22 - 2	0.83	0.75	0.88	0.86	0.90
Subject 23 - 2	0.83	0.75	0.88	0.86	0.90
Subject 24 - 2	0.83	0.75	0.88	0.86	0.90
Subject 25 - 2	0.83	0.75	0.88	0.86	0.90
Subject 26 - 2	0.83	0.75	0.88	0.86	0.90
Subject 27 - 2	0.83	0.75	0.88	0.86	0.90
Subject 28 - 2	0.83	0.75	0.88	0.86	0.90
Subject 29 - 2	0.83	0.75	0.88	0.86	0.90
Subject 30 - 2	0.83	0.75	0.88	0.86	0.90
Subject 1 - 3	0.81	0.65	0.91	0.86	0.91
Subject 2 - 3	0.83	0.70	0.91	0.86	0.90
Subject 3 - 3	0.83	0.75	0.88	0.85	0.89
Subject 4 - 3	0.81	0.70	0.88	0.84	0.90
Subject 5 - 3	0.81	0.70	0.88	0.84	0.90
Subject 6 - 3	0.81	0.65	0.91	0.86	0.91
Subject 7 - 3	0.81	0.70	0.88	0.84	0.90
Subject 8 - 3	0.81	0.65	0.91	0.86	0.91
Subject 9 - 3	0.81	0.65	0.91	0.86	0.91
Subject 10 - 3	0.81	0.65	0.91	0.87	0.91
Subject 11 - 3	0.81	0.70	0.88	0.84	0.90
Subject 12 - 3	0.79	0.65	0.88	0.82	0.87
Subject 13 - 3	0.79	0.70	0.85	0.85	0.90
Subject 14 - 3	0.81	0.65	0.91	0.86	0.91
Subject 15 - 3	0.77	0.65	0.85	0.86	0.90
Subject 16 - 3	0.81	0.70	0.88	0.84	0.90
Subject 17 - 3	0.81	0.75	0.85	0.85	0.89
Subject 18 - 3	0.81	0.65	0.91	0.86	0.91
Subject 19 - 3	0.81	0.65	0.91	0.86	0.91
Subject 20 - 3	0.81	0.65	0.91	0.86	0.91
Subject 21 - 3	0.81	0.65	0.91	0.86	0.91
Subject 22 - 3	0.81	0.65	0.91	0.86	0.91
Subject 23 - 3	0.81	0.65	0.91	0.86	0.91
Subject 24 - 3	0.85	0.70	0.94	0.87	0.91
Subject 25 - 3	0.85	0.70	0.94	0.87	0.91

Table S2: **Intersubject prediction analysis.** Performance using the rMPS in each individual subject's High ΔGE Regions as predictors.

Subject Number & Number of Regions	Accuracy	Sensitivity	Specificity	AUC-Testing	AUC-Training Average
Subject 26 - 3	0.81	0.65	0.91	0.86	0.91
Subject 27 - 3	0.81	0.70	0.88	0.84	0.90
Subject 28 - 3	0.81	0.65	0.91	0.86	0.91
Subject 29 - 3	0.87	0.80	0.91	0.88	0.93
Subject 30 - 3	0.81	0.65	0.91	0.86	0.91
Subject 1 - 4	0.79	0.70	0.85	0.83	0.90
Subject 2 - 4	0.81	0.70	0.88	0.85	0.90
Subject 3 - 4	0.83	0.70	0.91	0.83	0.91
Subject 4 - 4	0.85	0.70	0.94	0.86	0.91
Subject 5 - 4	0.75	0.65	0.82	0.83	0.90
Subject 6 - 4	0.75	0.65	0.82	0.83	0.90
Subject 7 - 4	0.75	0.65	0.82	0.83	0.90
Subject 8 - 4	0.75	0.65	0.82	0.83	0.90
Subject 9 - 4	0.75	0.65	0.82	0.83	0.90
Subject 10 - 4	0.85	0.75	0.91	0.86	0.92
Subject 11 - 4	0.75	0.65	0.82	0.83	0.90
Subject 12 - 4	0.81	0.65	0.91	0.86	0.91
Subject 13 - 4	0.79	0.70	0.85	0.85	0.91
Subject 14 - 4	0.75	0.65	0.82	0.83	0.90
Subject 15 - 4	0.75	0.60	0.85	0.85	0.90
Subject 16 - 4	0.75	0.65	0.82	0.83	0.90
Subject 17 - 4	0.87	0.80	0.91	0.86	0.95
Subject 18 - 4	0.75	0.65	0.82	0.83	0.90
Subject 19 - 4	0.75	0.65	0.82	0.83	0.90
Subject 20 - 4	0.81	0.70	0.88	0.86	0.91
Subject 21 - 4	0.85	0.75	0.91	0.87	0.95
Subject 22 - 4	0.85	0.75	0.91	0.86	0.92
Subject 23 - 4	0.75	0.65	0.82	0.83	0.90
Subject 24 - 4	0.81	0.70	0.88	0.86	0.91
Subject 25 - 4	0.81	0.70	0.88	0.86	0.91
Subject 26 - 4	0.85	0.75	0.91	0.87	0.93
Subject 27 - 4	0.75	0.65	0.82	0.83	0.90
Subject 28 - 4	0.75	0.65	0.82	0.83	0.90
Subject 29 - 4	0.85	0.75	0.91	0.87	0.93
Subject 30 - 4	0.75	0.65	0.82	0.83	0.90
